# Supplementary material for: Large language model based framework for automated extraction of genetic interactions from unstructured data
Source: PLoS One. 2024 May 21;19(5):e0303231. doi: 10.1371/journal.pone.0303231 (PMC11108146; doi:10.1371/journal.pone.0303231)
Supplement: Attachment — Submitted filename: Review-PONE-D-23-31346-From Bidhu K Mohanti.docx [file pone.0303231.s001.docx]

Review-PONE-D-23-31346

To Editor: The authors need to revise the paper, to make it concise and comprehensible for a reader of the journal. Separate suggestion and guidance as per review has been given for the Authors.

To Authors:

General:

1.Overall text content should be revised, edited and shortened. Similarly the references which are relevant should be cited. Example: For such a very specific objective/aim of the paper, Introduction is too lengthy, more than 1400 words with 22 references.

2. Authors should keep in mind to revise to make each section concise and comprehensible.

Specific:

1. Introduction-to outline the Aims and Objectives of this paper for a reader.
2. Back ground literature should concentrate on current AI approaches which can facilitate data extraction beyond the Key words and subject headings(e.g. MeSH) for data mining.
3. Methods: should explicitly focus on pre-and post-processing, followed by the proposed confidence factor with examples from the published literature on the subject headings.
4. Results: the authors extend the argument with benchmark datasets and real-world database that GIX (Gene Interaction Extraction) should become the overarching approach. Is this the overall outcome derived?
5. Conclusion and Abstract should give homogenous statement (e.g. Sentence in Abstract, “With suitable experiments, we show GIX's capability to augment existing datasets with new sentences”).Thus GIX appears to be a tentative step.
