# Supplementary material for: Large language model based framework for automated extraction of genetic interactions from unstructured data
Source: PLoS One. 2024 May 21;19(5):e0303231. doi: 10.1371/journal.pone.0303231 (PMC11108146; doi:10.1371/journal.pone.0303231)
Supplement: Attachment — Submitted filename: Response to Reviewers.docx [file pone.0303231.s002.docx]

**Manuscript ID:** PONE-D-23-31346

**Title:**

Large language model-based framework for automated extraction of genetic interactions from unstructured data

**Authors:**

Jaskaran Gill, Madhu Chetty, Suryani Lim, Jennifer Hallinan

We thank the editor and reviewers for their valuable insights and recommendations. All the suggested changes which were requested by the reviewers have been incorporated into the revised manuscript, thereby significantly improving its quality.

In response to the four queries mentioned under “Journal requirements”, we need to inform all these queries have been answered while addressing the reviewers. The supporting information file (S1 Supplementary Data) is now available through Github at <https://github.com/JaskaranKaurGill1/Gene-Interaction-Extraction.git>. Hence, the files should be accessible.

**Response to Reviewers’ comments**

Reviewer 1

Their method noticeably outperforms all competing methods. This is a vital and interesting work. The manuscript is well-written, organized, and structured.

***Major Comments:***

**Query 1.** The three queries below relate to the common concern about “bias”. Hence these are taken together as there is a common response to explain the queries.

a) *It needs to be clarified if there is an overlap between the sentences in the training set and the articles/text used as evidence for gold-standard databases. If there is overlap, this creates bias due to information leakage, and a remedy needs to be applied.* *Similarly, what about the overlap between sentences in/used in datasets such as BioInfer, etc, vs. RegulaonDB? If yes, this creates bias due to information leakage. Therefore, either a clarification is needed, or a remedy needs to be applied.*
b) *Additionally, the same datasets used for testing should not be used for finetuning LLMs as this introduces bias. A remedy must be applied to improve the reliability of the results.*
c) *It needs to be clarified how the hyperparameters, such as the number of epochs, were tuned. Was it 10-fold CV using only the train data? If not, there is bias. So, either a clarification is required, or a remedy is necessary to improve the reliability of the results. At the very least, 10-fold nested cross-validation must be used.*

**Response to Query 1.** We regret the confusion and thank the reviewer for highlighting and seeking clarification about this important issue. Indeed, as the reviewer correctly pointed out, we fully agree that the testing dataset should be completely independent of the fine-tuning data sets. In other words, the data used for testing should not be part of training process, else, as the reviewer has stated, it will introduce bias.

Foremost, we explicitly clarify that in none of the experiments has any occurrence of data overlap or information leak. This can be clarified as follows.

BioBERT, being pre-trained and empowered with the contextual understanding of biomedical text, only requires fine-tuning to perform specific tasks such as named entity recognition and relation extraction. In GIX, two pre-trained BioBERT models are being fine-tuned, first is the Sentence Eliminator 1 in pre-processing stage (Stage-1) and the second is the Relation Classifier in relation extraction stage (Stage-2). The fine-tuning of these two BioBERT models are conducted using “training” dataset, while a wholly independent “testing” dataset is specifically set aside for evaluation purposes. The allocation of which datasets are used for fine-tuning and which one for testing varies, based on the specific experiment being conducted, as explained below.

- Exp 1: Relation extraction capability (Stage-2) of the GIX framework

This experiment assesses the performance of GIX-RE (Stage-2) against state-of-the-art methodologies. We applied 10-fold cross-validation with each of the four benchmark datasets (HPRD50, BioInfer, IEPA and LLL). Experiments are carried out for each dataset individually by dividing it into 10 equal folds. For each fold, the fine-tuning is done from scratch using the baseline BioBERT model (original setting) with the 9 folds and the testing is carried out with the 10^th^ fold. In each iteration, using a different fold as the test set ensures comprehensive evaluation across diverse data samples, contributing to the model's robustness and generalisability. The final accuracy reported is the average results obtained from the 10 individual experiments (folds) for each dataset. In fact, we wish to clarify that to ensure a fair and meaningful comparison to other state-of-the-art methods, we followed exactly the same process.

To provide clarity to the readers and avoid confusion, the following text is inserted in revised manuscript (Section: Exp1- Relation extraction capability (Stage-2) of the GIX framework page 15).

To evaluate a model’s performance and generalisation ability, we employed the widely used 10-fold cross validation provided by the KFold library from Scikit-Learn [1] to each of the four datasets (HPRD50, BioInfer, IEPA and LLL). In brief, the method involves dividing the dataset into 10 equal subsets, with 9 subsets used for fine-tuning and the isolated 10^th^ subset used for testing. For each dataset, the process is repeated 10 times using a different fold as the test set, and each time, the relation classification model gets fine-tuned from scratch (original setting). The overall accuracy is determined by averaging the results from the 10 individual experiments (folds) conducted on each dataset. This ensures comprehensive evaluation across diverse data samples, contributing to the model's robustness and generalisability. This approach has been commonly used in several state-of-the-art methods in different domains including Biological relation extraction, e.g. Bi-LSTM [2] , MCCNN [3], GK [4], NHGK [5], EDG [6], PIPE [7], WWSK [8], RCNN [9], DNN [10], RNN + CNN [11] and iLSTM+tAttn [12] .

- Exp 2: Comparison of GIX with manual curation of a benchmark dataset

In this experiment, we used LLL benchmark dataset, which contains relationships related to *Bacillus subtilis*, for evaluating how well the proposed GIX model performs vis-à-vis the manual curation process. The datasets used for fine-tuning the classifier models in GIX stages 1 and 2 are BioInfer, HPRD50, and IEPA while the sentences from independent LLL dataset are used for testing.

To provide clarity to the readers and avoid confusion, the following text is inserted in revised manuscript (Section: Exp2- Comparison of GIX with manual curation of a benchmark dataset, page 17)

In GIX, fine-tuning is required for both the Sentence Eliminator 1 (Stage-1) and Relation Classification (Stage-2), as they utilise a variant of the BioBERT model. The fine-tuning is performed using sentences from the three datasets(HPRD50, BioInfer, and IEPA), while the testing is performed on the independent LLL dataset.

- Exp 3: Comparison of GIX with manual curation of a real-world dataset

In Exp 3 related to GIX vs. RegulonDB, all four benchmarks (BioInfer, HPRD50, LLL, and IEPA) datasets are used for fine-tuning. Testing was conducted on a completely independent dataset containing *E. Coli*-related sentences. These sentences pertaining to *E. Coli* are extracted from published literature during GIX Stage-1, and thus, do not intersect with any sentences found in the four datasets used during fine-tuning. The fine-tuned GIX model is applied to the extracted “unseen” dataset to compare GIX performance with RegulonDB, which is the ground truth. RegulonDB is a real-world database curated manually.

To provide clarity to the readers and avoid confusion, the following text is inserted in revised manuscript (Section: Exp3- Comparison of GIX with manual curation of a real-world database, page 20).

The datasets used for fine-tuning the Sentence Eliminator-1 (Stage-1) and the Relation Classification (Stage-2) are the four available benchmark datasets, i.e., BioInfer, HPRD50, IEPA and LLL. The testing dataset was formulated using sentences not found in the fine-tuning data sets. These sentences were extracted by GIX through keyword-based extraction from PubMed. It may be noted that the sentences comprising the testing dataset, obtained from published literature, are related to *E. Coli*, while the benchmark datasets used for fine-tuning are from different domains. For example, IEPA dataset is focused on biochemical relations and the LLL is dedicated to *Bacillus subtilis*. Thus, there is no overlap between the content of these four datasets and the *E. Coli*-related sentences used for testing.

**Query 2:** *On a similar note, how were the rest of the decisions about the pipeline made? For example, finding optimal values for parameters like alpha and K. Was it only using training data? If not, there is bias. So, either a clarification is required, or a remedy is needed to improve the reliability of the results.*

**Response to Query 2:** The choice of the balancing parameter, K, in Equation 1 relies on the observed values of CF_1_ and CF_2_, occurring after the completion of the relation extraction stage. Therefore, it is independent of the fine-tuning process.

The values of parameter K depend on the datasets being used:

- For the *Exp1*- *Comparison of GIX with manual curation of a* ***benchmark dataset*** experiment, K=1
- For the *Exp2*- *Comparison of GIX with manual curation of a* ***real-world dataset*** experiment, K=2

For our work, the value of parameter K was determined by examining the distribution of CF_1_ and CF_2_. And choosing a value that provides the necessary balance between these two components. Alternatively, as the reviewer has suggested, a more rigorous experimentation could be carried out with various values of K and an optimal value decided. As the focus of the current research work is on automated extraction of gene work, current method for setting may be considered sufficient. The need for investigating optimal setting for K parameter is important and can be pursued as future extension to the work.

Equation two has been revised to eliminate α. This modification has been made with the aim of simplifying the equation, enhancing its clarity, and facilitating comprehension for users. This adjustment is in response to query 8 raised by reviewer 3.

***Minor Comments:***

**Query 3.** There are a few typos and grammatical errors. Please proofread or use an editing service like Grammarly. The last sentence on page 6 requires a supporting citation. Line 413 mentions “significantly”. So, either provide p-values or avoid using that term.

**Response to Query 3**: Thank you for raising this. We have used Grammarly and a professional editor has also proof-read the revised manuscript. Citation is provided to last sentence on page 6. As suggested, the term “significantly” has now been deleted from the sentence.

**Query 4.** *In table 5, it is unclear whether the repeated performance for GIX-RE and GIX(full) is a typo or not.*

**Response to Query 4:** We clarify that the identical performance for GIX-RE and GIX (full) is not an error. In fact, we anticipate them to be identical. This is because we aim to demonstrate that GIX, utilizing a few target-related keywords (a simpler approach), instead of manually collecting, refining, and annotating sentences (more complicated and cumbersome approach), continues to maintain a same accuracy in extracting interactions without losing crucial information.

The following revised text is inserted in Section: Exp-2 Comparison of GIX with manual curation of a benchmark dataset on Page: 19.

Table 5 (of the manuscript) displays the results of two experiments labeled as Exp2-(i) GIX-RE (only relation extraction model) and Exp2-(ii) GIX (Full Framework). In experiment Exp2- (i), only the relation extraction model of GIX, corresponding to Stage-2 of the framework, is utilised. The datasets used for fine-tuning of the relation classification model for experiment Exp2-(i) include HPRD50, IEPA, and BioInfer while LLL dataset is used for testing. In experiment Exp2- (ii), the full GIX framework is under investigation. Here, the fine-tuning datasets consist of HPRD50, IEPA, and BioInfer. While the testing dataset comprises of sentences extracted from PubMed related to *Bacillus Subtilis* regulatory interactions. These two experiments demonstrate that GIX, using just a few target-related keywords instead of the manually refined and formatted sentences was able to maintain a similar level of accuracy of extracted interactions without losing any of the sentences.

Reviewer 2:

The manuscript definitely represents the interest for Plos One readership. However, I have some comments and suggestions.

**Query 1.** *Specify more transparently what are the stages that are used for labelling sentences that do not include relations in the training set.*

**Response to Query 1.** We regret if there is any ambiguity regarding the stages and the labelling of sentences. The labelling process occurs during Stage-2 of GIX. To elaborate, all four datasets being used for fine tuning, namely LLL, BioInfer, HPRD50 and IEPA, consist of sentences having a minimum of two gene/protein entities within each sentence. This is true for every sentence within each dataset, irrespective of the relationship between the entities. The sentence labelling procedure, involving the replacement of the gene/protein pair under consideration with labels $GENE_AGENT# and $GENE_TARGET#, remains consistent across all sentences. For sentences extracted from the published literature, those lacking a minimum of two entities are excluded by sentence eliminator 2 during the Stage-1 of GIX.

The following revised text is now inserted in the Section: Relation extraction on page 9.

As a result of sentence eliminator-2, which filters out sentences with fewer than two gene/protein entities, the sentences undergoing processing for relation classification invariably contain a minimum of two gene/protein entities, irrespective of the presence or absence of an actual relationship. The labelling criteria remain consistent for all sentences before relation extraction. To extract binary relations, each entity pair in a sentence is substituted with a label to clearly identify both the agent and target entities involved in the relationship. The first entity of a pair is replaced with $GENE_AGENT# and the latter is substituted with $GENE_TARGET#.

**Query 2.** *Discuss in the text, which parameters/models provide the highest accuracy of relations extractions in imbalanced data sets (scientific abstracts and publications are, by definition, imbalanced data sets).*

**Response to query 2:** We agree with the reviewer's observation regarding the imbalanced nature of datasets for scientific literature, particularly, in the context of biological relation extraction. In this work, the highest accuracy of relation extraction model (Reported in Table 3 of the manuscript) is achieved by implementing the GIX framework with the hyperparameters given in Table 1 of the manuscript. The reported hyperparameters were selected by thorough experimentation by applying 10-fold cross-validation to ensure robustness generalisability.

To clarify the role played by GIX framework in addressing the relation extraction issue within imbalanced datasets, we have inserted the following text in the revised manuscript in Section: Methods on page 7.

In a sentence with 2 entities, the number of potential relationships, taking direction into consideration is two. As the number of entities in a sentence increase, the possible relationships amongst them can grow exponentially. However, the number of true relationships does not grow at a similar pace, thereby, leading to a situation when the number of non-interactions far exceed the number of interactions and resulting to imbalance dataset. The pre-processing (Stage-1) and post-processing stages (Stage-2) in GIX are designed to recognise non-relation entity pairs, to decrease the number of sentences classified as true negatives. This reduces the gap between the two classes, which contributes to the imbalance, thereby enhancing the overall performance and accuracy of relation extraction in BioBERT.

**Query 3.** *How does the algorithm identify relations that are not provided in the sentences explicitly but can be supposed using the semantic analysis made by experts during manual annotation.*

**Response to query 3:** We appreciate the reviewer for bringing up an intriguing point. BioBERT, the relation classifier used in GIX, is a transformer-based model pretrained on biological corpus. Transformer-based models can identify complex relationships by leveraging contextual embeddings and pre-trained representations to capture semantic information within sentences. The attention mechanism allows the model to weigh the importance of words in a sentence, capturing complex dependencies. Transformer models, pre-trained on relevant corpus, learn domain-specific language patterns, terminologies, and contextual information that are crucial for tasks within the biological domain.

To ensure clarity to the readers, the manuscript has been revised and following text is inserted in Section: Relation extraction, page 9.

Through fine-tuning on expert-annotated data, pre-trained models efficiently transfer general language knowledge to domain-specific tasks, resulting in improved task performance and adaptability.

**Query 4.** *Please provide the code of the models in the Supplementary Materials.*

**Response to query 4:** Thank you for raising this issue related to availability of source code. While we are happy to include the code, it is unclear whether the reviewer is suggesting a language specific source code (Python based in our case) or general pseudocode to be included in the supplementary material. In our humble opinion, providing a pseudocode, along with the detailed step-by-step explanation, may prove more useful since it is a language-independent and generic representation. Using the pseudocode, the readers can then decide their preferred programming languages to implement the pseudocode . However, if there is a specific requirement for the source code, it can be readily provided. Currently, we have included the pseudocode and Python source code in a public repository accessible at https://github.com/JaskaranKaurGill1/Gene-Interaction-Extraction. In addition to the code, we have also shared the processed datasets and models used for fine-tuning and testing the relation extraction and sentence eliminator BioBERT model. Needless to say, the availability of both the pseudo-code and datasets will assist the readers in reproducing the methodology.

**Query 5.** *I suggest that the authors discuss in more details the obtained results of gene regulatory network built using the developed approach from the biological point of view.*

**Response to query 5:** We appreciate the reviewer for their valuable suggestion. It may be noted that there are several sophisticated gene regulatory models, based on, for example Bayesian, Fuzzy, non-linear differential equation and so on. All of these methods are highly computation intensive. They start with random networks and mostly rely on heuristic approaches for their solution. The section "Application of GIX: Gene Regulatory Network Inference" is aimed to demonstrate a direct application of the proposed research. As shown, the network inferred by the proposed research can be used as an initial “prior knowledge infused genetic network” for other sophisticated methods to be applied. This work is beyond the scope of current research and we intend to publish it as a separate research publication.

Having said this, in response to the suggestion, we are still able to incorporate a more in-depth discussion of the obtained results of the gene regulatory network constructed using our developed approach. The revised text is inserted in Section: ‘Application of GIX: Gene Regulatory Network Inference’ Page: 22 is as follows:

Biological circuits offer valuable insights into molecular-level interactions, especially within Gene Regulatory Networks (GRNs). From the presented GRN network *for E. coli*, entities *CRP, fnr, CytR, fis, MarA, csgD* stand out as the key regulatory genes, regulating 77 genes among themselves. Identifying controller genes is crucial as they govern gene expression, influencing cell state and offer help in developing targeted treatments for genetic disorders like cancer. GRNs exhibit sparsity, evident in the presented network where 334 genes exhibit only 500 interactions. Further, it may also be noted that due to the high cost of wet lab experiments to determine interactions, exhaustive exploration of relationships among thousands of genes becomes impractical. While significant efforts have been made to develop advanced computational methods for inferring relationships using gene expression data, yet the noisy and scarce nature of the data poses several challenges in achieving improved accuracy and efficiency. Integrating relationships obtained via GIX as a-priori knowledge in the reconstruction process can significantly reduce convergence time, enhance overall accuracy, and facilitate the discovery of new relations. This iterative process, once refined, allows for cost-effective high-throughput experiments targeting specific entities, reducing the overall expense of uncovering crucial relationships.

Reviewer 3

**General Comment:** I think the work needs to be completely rewritten, starting from a clear description of the main contributions and why the authors consider it to be original, and if the main objective is to produce a tool, give a clear and complete description of how to build and use the tool, preferably with available code to try it. Also, there are many methodological issued in the experimental section that rise doubts in the validity of the results.

**General Response:** Thank you for taking the time to provide valuable feedback on our manuscript. We appreciate your thorough review and constructive comments. We understand the importance of presenting a cohesive and easily understandable framework, and we have addressed these issues as well as clarifications related to the doubts about validity of the results.

As observed explicitly by reviewer 1 and reviewer 2, we wish to further clarify that, in our opinion, the paper makes a significant contribution and proposes a novel, systematic, and fully automated framework for robust genetic relation extraction, that efficiently identifies relevant publications with minimal keywords, optimizes sentence selection, and proposes a novel confidence factor for quantifying the reliability of extracted biological relations. While individual tools and technologies may evolve or change over time, our proposed well-designed framework providing the underlying structure, methodology or approach will remain ubiquitous. The novelty of a framework lies in its conceptualization, design principles, and how it orchestrates the integration of various tools to achieve the goal of biological relation extraction.

Regarding availability of source code, we agree with the suggestion of making this source code available, a detailed explanation regarding this is already provided on Page 5 of this document, in response to a similar query (4) from reviewer 2.

Specific Comments

**Query 1.** *Sentence in lines 88-90 need to be revised. I think it should say “are due” instead of just due.Line 95. Says “BERT and ELMo, one of”, they are two, not one. Lines 116-117, it says “For the selection of abstracts, we use of task-specific…”, should be “… we make use of task-specific…”*

**Response to Query 1**: We regret the inadvertent typos creeping in these lines. The manuscript has been fully proof-read and revised to expunge all such errors.

**Query 2.** *Line 215 on, section Pre-processing. It is not clear how the abstracts were pre-processed. Which search tool was used? How the keywords were used to filter the list of abstract candidates? Someone wanting to reproduce this work will need this information*

**Response to Query 2:** Again, we regret the perceived issues of clarity and the arising confusion. We have revised the Pre-processing section by providing detailed information about the tools and their application in extracting abstracts. To assist readers to reproduce the work, we have provided the data and the code in the supplementary material, including the code for pre-processing.

Further, for clarity to the readers, the following revised text is inserted in Section: Pre-processing, page: 8.

The Bio.Entrez module facilitates data retrieval from PubMed, a comprehensive biomedical literature database [13]. To find relevant PubMed articles, we crafted a search query using PubMed Bio.Entrez utilities. The PubMed search query requires a set of keywords and specifies the maximum number of documents to retrieve, resulting in a list of PubMed IDs ranked by relevance according to the provided keywords. With the retrieved PubMed IDs, we used the web scraping tool BeautifulSoup [14], a Python library, to extract titles and abstracts from the papers on PubMed. The chosen set of keywords used for extraction gene regulation relationships is outlined in the experiment section under "Selection of Keywords."

Moreover, as requested by the reviewer, we have also updated Figure 1 to incorporate the relevant tools used at each step in the GIX processes.

Figure 1: Schematic of fully automated relation extraction using an LLM based GIX framework where the white blocks signify the processes, and the grey blocks indicate the tools utilized within those processes.

**Query 3.** *Line 227, Sentence Tokenization: It is not clear how Biobert and BERN2 are used to eliminate sentences that are not relevant. What do you input the models? Do you need to fine tune them also? How is this performed?*

**Response to Query 3:** Based on reviewers query, we have revised the Sentence Tokenization section to make it explicitly clear that the removal of sentences does not take place during the sentence tokenization but after sentence tokenization. Further clarification regarding the input and process of sentence elimination using BioBERT and BERN2 is also provided. We have also clarified the fine-tuning process for each experiment in response to Query No. 4.

The following text is inserted in the revised manuscript in Section: Sentence-tokenization on page 9.

We split the final set of abstracts into individual sentences using Punkt sentence tokeniser from the Natural Language Toolkit (NLTK) [15]. Punkt is a pre-trained unsupervised machine learning model designed for detecting sentence boundaries. Then the tokenized sentences undergo two consecutive sentence elimination processes: the first utilises a fine-tuned BioBERT (biobert_v1.1_pubmed) classification model, and the second involves BERN2, as detailed in the subsequent sections.

The following revised text is inserted in Section: Sentence Eliminator 1 on page 9.

Sentence eliminator 1 identifies and removes sentences that do not discuss a regulatory interaction. A fine-tuning dataset was created from annotated PPI corpus sentences. The specific datasets used for fine-tuning and testing in each experiment are outlined in their respective sections. To create the fine-tunning dataset, sentences dictating at least one regulatory relationship receive a positive classification label, while the rest are labelled as negative. Once finetuned, the BioBERT model evaluates test sentences, assigning a classification of 0 if there are no genetic interactions or if the sentence discusses non-regulation, and 1 if the sentence contains a relational context of a gene/protein interaction. Sentences classified as 0 are eliminated, while those classified as 1 undergo further evaluation for the presence of named entities.

The following revised text is inserted in Section: Sentence Eliminator 2 on page 9.

For the second sentence elimination step, since at least two entities are involved in a regulatory relationship, all sentences that contain fewer than two gene or protein entities are excluded. This elimination is carried out using BERN2, without any additional fine-tuning, to recognise gene and protein entities in a sentence. BERN2 can be implemented through their RESTFUL AP [16] I. For each sentence, BERN2 produces a JSON list of annotations, containing the entity phrase, its entity type, and a probability score. Sentences are eliminated if the annotation list contains fewer than two gene/protein entities.

**Query 4.** The three queries below relate to the common concern about fine-tuning process of the experiment: Exp1: Relation extraction capability (Stage-2) of the GIX framework. Hence these are taken together since there is a common response to explain the queries.

a. *Line 237, sentence eliminator 1: It is not clear how the fine tune is performed. And moreover, you are using the sentences in the 4 datasets you will use in the experiments to test the method, to fine-tune part of the method, in this way, the final test will not be independent of the training. Another very important issue with this experiment is that Stage 1 of the proposed method includes the finetuning of BioBERT with the same 4 datasets used in this experiment to evaluate stage 2. In this way, even if the evaluation was done by leaving one dataset out (which was not clear, as already told two comments above), parts of the test set have been used for training (of stage 1), thus making invalid those results as the test dataset are no longer independent of the training set.*
b. *Line 411, data on Table 3. It is not clear how the evaluation for alternative methods was obtained. Does the authors of the manuscript performed all test over the same datasets and in the same conditions for all the cases? Or they just take the value of some experiment in another work? If it is the second case the results are not directly comparable, as the experimental condition may have been different for each experiments, even using dependent data or overlapping data between train and test. This should be clarified very carefully and may invalidate the comparison.*
c. *Line 398, Relation extraction capabilities It is not clear how the validation experiments are performed. It says that the method was repeated 10 times using a different dataset for validation, but how exactly is this done? If there are 4 datasets and you leave one dataset out for validation, you can make only 4 repetitions. And also, it is not clear what is meant by training, what is trained and how? You should clearly explain how you run your experiments, for both, making it easy to reproduce your results, and checking if the experimental procedures was correct, or if you are using the same information for training and test. In the way it is written now, it makes impossible to reproduce the work, and rises concerns about the independence of the data used in training and test.*

**Response to Query 4:** We regret the confusion. As the reviewer has correctly emphasized, we acknowledge the importance of ensuring complete independence between the testing dataset and the fine-tuning datasets. Needless to say, it is crucial to avoid any overlap between the data used for testing and the data for the fine tuning (training) process to prevent any bias.

We explicitly state that in none of the experiments reported here, any data overlap or information leak occurred. In response to a similar query (Query 1) from Reviewer 1, we have already provided a thorough clarification earlier. It is repeated again below.

BioBERT, being pre-trained and empowered with the contextual understanding of biomedical text, only requires fine-tuning to perform specific tasks such as named entity recognition and relation extraction. In GIX, two pre-trained BioBERT models are being fine-tuned, first is the Sentence Eliminator 1 in pre-processing stage (Stage-1) and the second is the Relation Classifier in relation extraction stage (Stage-2). The fine-tuning of these two BioBERT models are conducted using “training” dataset, while a wholly independent “testing” dataset is specifically set aside for evaluation purposes. The allocation of which datasets are used for fine-tuning and which one for testing varies, based on the specific experiment being conducted, as explained below.

- Exp 1: Relation extraction capability (Stage-2) of the GIX framework

This experiment assesses the performance of GIX-RE (Stage-2) against state-of-the-art methodologies. We applied 10-fold cross-validation with each of the four benchmark datasets (HPRD50, BioInfer, IEPA and LLL). Experiments are carried out for each dataset individually by dividing it into 10 equal folds. For each fold, the fine-tuning is done from scratch using the baseline BioBERT model (original setting) with the 9 folds and the testing is carried out with the 10^th^ fold. In each iteration, using a different fold as the test set ensures comprehensive evaluation across diverse data samples, contributing to the model's robustness and generalisability. The final accuracy reported is the average results obtained from the 10 individual experiments (folds) for each dataset. In fact, we wish to clarify that to ensure a fair and meaningful comparison to other state-of-the-art methods, we followed exactly the same process.

To provide clarity to the readers and avoid confusion, the following text is inserted in revised manuscript (Section: Exp1- Relation extraction capability (Stage-2) of the GIX framework page 15).

To evaluate a model’s performance and generalisation ability, we employed the widely used 10-fold cross validation provided by the KFold library from Scikit-Learn [1] to each of the four datasets (HPRD50, BioInfer, IEPA and LLL). In brief, the method involves dividing the dataset into 10 equal subsets, with 9 subsets used for fine-tuning and the isolated 10^th^ subset used for testing. For each dataset, the process is repeated 10 times using a different fold as the test set, and each time, the relation classification model gets fine-tuned from scratch (original setting). The overall accuracy is determined by averaging the results from the 10 individual experiments (folds) conducted on each dataset. This ensures comprehensive evaluation across diverse data samples, contributing to the model's robustness and generalisability. This approach has been commonly used in several state-of-the-art methods in different domains including Biological relation extraction, e.g. Bi-LSTM [2] , MCCNN [3], GK [4], NHGK [5], EDG [6], PIPE [7], WWSK [8], RCNN [9], DNN [10], RNN + CNN [11] and iLSTM+tAttn [12] .

- Exp 2: Comparison of GIX with manual curation of a benchmark dataset

In this experiment, we used LLL benchmark dataset, which contains relationships related to *Bacillus subtilis*, for evaluating how well the proposed GIX model performs vis-à-vis the manual curation process. The datasets used for fine-tuning the classifier models in GIX stages 1 and 2 are BioInfer, HPRD50, and IEPA while the sentences from independent LLL dataset are used for testing.

To provide clarity to the readers and avoid confusion, the following text is inserted in revised manuscript (Section: Exp2- Comparison of GIX with manual curation of a benchmark dataset, page 17)

In GIX, fine-tuning is required for both the Sentence Eliminator 1 (Stage-1) and Relation Classification (Stage-2), as they utilise a variant of the BioBERT model. The fine-tuning is performed using sentences from the three datasets(HPRD50, BioInfer, and IEPA), while the testing is performed on the independent LLL dataset.

- Exp 3: Comparison of GIX with manual curation of a real-world dataset

In Exp 3 related to GIX vs. RegulonDB, all four benchmarks (BioInfer, HPRD50, LLL, and IEPA) datasets are used for fine-tuning. Testing was conducted on a completely independent dataset containing *E. Coli*-related sentences. These sentences pertaining to *E. Coli* are extracted from published literature during GIX Stage-1, and thus, do not intersect with any sentences found in the four datasets used during fine-tuning. The fine-tuned GIX model is applied to the extracted “unseen” dataset to compare GIX performance with RegulonDB, which is the ground truth. RegulonDB is a real-world database curated manually.

To provide clarity to the readers and avoid confusion, the following text is inserted in revised manuscript (Section: Exp3- Comparison of GIX with manual curation of a real-world database, page 20).

The datasets used for fine-tuning the Sentence Eliminator-1 (Stage-1) and the Relation Classification (Stage-2) are the four available benchmark datasets, i.e., BioInfer, HPRD50, IEPA and LLL. The testing dataset was formulated using sentences not found in the fine-tuning data sets. These sentences were extracted by GIX through keyword-based extraction from PubMed. It may be noted that the sentences comprising the testing dataset, obtained from published literature, are related to *E. Coli*, while the benchmark datasets used for fine-tuning are from different domains. For example, IEPA dataset is focused on biochemical relations and the LLL is dedicated to *Bacillus subtilis*. Thus, there is no overlap between the content of these four datasets and the *E. Coli*-related sentences used for testing.

**Query 5.** *Lines 472 to 480. The discussion in this paragraph is hard to unde3rstand and should be rewritten. But I think I understand that it states that if you finetune properly the system without using any information from the test dataset (as I suggested in previous comments) then the performance of the system will be reduced. If this is what was meant, I must say this statement is obvious and is what I keep saying in my comments, that the validation was not done with an independent dataset, and thus is invalid. And if the performance is reduced, it means that the system cannot generalize, so it will be of no use to discover new relations, only to reinforce already known relations. I think this issue is key and should be clearly discussed and addressed, which was not properly done in this manuscript.*

**Response to Query 5:** We appreciate the reviewer for bringing to attention this important matter. As discussed in Query 4, all our validation datasets were kept independent of fine-tuning datasets.

In the case of Exp2, the model was fine-tuned using non-LLL datasets and tested on LLL. The decrease in accuracy is very small, more important issue that our model still outperforms other models. It may be noted that the generalisation is achieved by using a large dataset for fine tuning: 10,916 sentences by combining BioInfer, IEPA, and HPRD50.

To provide clarity to the readers, Lines 472 to 480 of section: Evaluation of automated extraction by GIX against a manually curated benchmark dataset have been revised as follows in the updated manuscript:

The use of non-related datasets in Exp2 is realistic because in the real-world, often, the data is not always truly aligned with the training dataset. In past, the attempts by researchers at generalisation have been less successful with their accuracies decreasing significantly. For instance, in [12], the model that was trained using only BioInfer and tested on LLL exhibited a low accuracy of 33.50%. In contrast, our proposed model has a Precision of 86.30% which is a slight decrease from Exp1 but still higher than other models. Combination of diverse non-related datasets (BioInfer, IEPA and HPRD50) has led to better generalisation and hence improved performance.

**Query 6.** *Another concern, you need to explain better how the positive class for a sentence is decided. From your description, it seems that the relation must be encoded completely in one sentence, but sometimes an author would use several sentences to describe a positive interaction, how is this managed? Line 246, Sentence eliminator 2: Again, you discard a sentence if there are less than 2 gene/protein present in the sentence. But sometimes the relations are explained in several sentences, with one entity in each. And again, it is not explained at all how this was performed (and in this case it is not even mentioned which tool (perhaps BioBERT?) was used).*

**Response to Query 6:** Thank you for highlighting an interesting point about relationships embedded within multiple sentences. In this study, our datasets for fine-tuning and testing exclusively involve relationships within individual sentences (intra-sentential), focusing on sentence-level relationships. Literature [2, 8, 6, 9, 5] on biological relations have also focussed on detecting single sentence relationships. Thus, the scope of the proposed research has been limited to identifying intra-sentential relationship. While most of the genetic interactions would normally appear within a sentence, as the reviewer has stated, there can be relationships (although limited) which can appear in multiple sentences. Further, difficulties also arise from the limited availability of annotated datasets for cross-sentential relation extraction in the biology domain. It is a significant amount of work and hence, the current research is focussed on intra-sentential relations and exploration of multi-sentential relationships is a potential avenue for future work. We have included this in the Section: Conclusion (page 23) as potential area of research.

**Query 7.** *Line 254, Relation Extraction: again, no details are given on how the fine-tuning of BERN2 and BioBert was done. Also, it is not clear to me why you need to replace the entity pair by generic labels. I imagine this can negatively affect the language model. The entity itself can carry context information useful for the language model. Imagine the word sequence “ The car is gray”, if I substitute car by a label $SUBSTANTIVE$ I am actually removing important information, as cars usually have a limited number of colors, and other substantives like “bears” have different sets of colors. So when you remove the real entity tag and replace it, you are removing contextual information useful for the language model. Thus, you need to clearly justify the need for this entity replacement.*

**Response to Query 7.** We would like to humbly digress from the reviewer’s viewpoint. To our understanding, the approach of substituting recognized entities with generic labels aligns with common practices in NLP relation extraction [15]. We again clarify that the purpose of the substitution which is to focus on the relationship patterns rather than the specific identities of entities. For example, consider the following:

Here, the crucial knowledge to extract from the original sentence pertains to the relationships between the entities *SigK* and *ykvP* rather than the entities themselves. To capture these relationships, these entities were replaced with meaningful labels for identification, namely $GENE_AGENT# and $GENE_TARGET#. It is noteworthy that these labels not only retain the type of entity but also specify the directional relationship from agent to target. The inclusion of the $ and _ in these labels serves to make them stand out in the sentences, facilitating a clearer interpretation for the model.

It is important to note that when a sentence involves more than two entities, generating multiple versions of the sentence with entities replaced by generic labels such as "BLANK" proves effective in capturing relationships between different pairs of entities. We experimented with several substitution words—GENE, ENTITY, and BLANK—and observed that BLANK yielded the highest accuracy. This observation might be attributed to the contextual meanings associated with the other words in the biological domain.

Figure 2 below illustrates another example of the relation extraction process proposed in our work. In the relation extraction process using the proposed GIX-RE (Stage-2) for directed binary relationships, the NER model detects three entities: GeneX, GeneY, and GeneZ, generating a potential of 6 relationships. Six sentence variants are generated, each highlighting a specific relationship through replacement labels $GENE_TARGET# and $GENE_AGENT#. These labelled sentences undergo relation classification, where the model accurately classifies true relationships as 1 and dismisses false relations by assigning a classification of 0.

Figure 2: Relation extraction process using proposed GIX-RE (Stage-2) for directed binary relationships.

**Query 8:** *The value of alpha is set depending on hoy much emphasis is assigned to component P, but that is the role of constant K, which balances the important of the two terms, it is not clear why there is a need to set an extra parameter alpha that will be given later a value of 1 (without exploring its optimal value in any sense). The same happens to constant K, which is later given a value of 1 without exploring optimal values.*

**Response to query 8:** Thank you for raising this.

Parameter K:

${CF}_{e_{a}e_{t}}=\sum_{s=1}^{n} v_{s}+K\sum_{T=1}^{M} P_{e_{a}e_{t}}^{T}$	(1)

$${CF}_{1}+K*{CF}_{2}$$

Indeed, as the reviewer correctly points out, the parameter K is for balancing the two terms CF_1_ and CF_2_. K parameter can adopt values depending on the datasets being used. For our work, the value of parameter K was determined by examining the distribution of CF_1_ and CF_2_. And choosing a value that provides the necessary balance between these two components. In our experiments, the value of K is either 1 or 2. For Exp2 ( *Comparison of GIX with manual curation of a benchmark dataset)*, it takes the value of 1. For Exp3 (*Comparison of GIX with manual curation of a real-world dataset)*, it is given the value of 2. The reason the value of K is higher in Exp3 than Exp2 is that the computed CF_1_ values of relations extracted in Exp3 are higher than that in Exp2. Therefore, to ensure the balance between the two terms, K=2 is used for Exp3. As the focus of the current research work is on automated extraction of gene work, the current method for setting K has been considered sufficient. However, as reviewer suggests, a more rigorous experimentation could be carried out with various values of K and an optimal value decided. We agree that, as future extension to the work, investigating the optimal setting for K parameter can be pursued.

Parameter α:

While K serves as a balancing factor between CF_1_ and CF_2_ in equation (1), the intended purpose of parameter α is to emphasize the relative emphasis between different scenarios of the prior knowledge component depicted by Equation 2. Including the constant α allows us to change the relative emphasis of different conditions of $P_{e_{a}e_{t}}$ shown in Equation 2. Although, in our studies, we could achieve the required distinction of different conditions of $P_{e_{a}e_{t}}$ by α=1, for sake of generalisation, we had decided to provide flexibility by incorporating α in Equation 2. Realising that this may cause confusion to the readers, we have now decided to remove α from the equation and make it much simpler as follows

$P_{e_{a}e_{t}}=\left\{ \begin{aligned} 1 &e_{a} is known agent but {relation e}_{a}e_{t} is unknown \\ 3 &e_{a}e_{t} is known relation \\ 0 &otherwise \end{aligned} \right.$	(2)

Regarding optimal setting for α, again, in our opinion, as the focus of the current work is on developing proof of concept for mechanism related to automated extraction of gene interactions, we would like to propose that this choice of settings should be acceptable and the investigations for identifying the optimal setting of α can be part of the future work. However, having said this, if it is felt that these experiments are necessary, that can be carried out and included.

**Query 9.** *Line 320, equation (1): All variables should be defined immediately after the equation. It is difficult to understand several lines of discussion and explanation of why the equation will work without knowing what the variables are. For example, variable v is defined in line 327(and is used ambiguously as in the equation it appears as v_s and in the explanation as just v)*

**Response to query 9:** We regret this omission on our part regarding keeping the variable definition in Equation 1 close to the equation. We have updated the Confidence Factor section to provide immediate definitions for all variables Further, as suggested, we have ensured that all references to variables are consistent (including, e.g., 'v_s_' and ‘v’) throughout the manuscript.

The proposed new measure, Confidence Factor ${CF}_{e_{a}e_{t}}$ between each entity pair *e_a_e_t_*, indicates the likelihood of existence of each of the interactions identified as existing between the agent/controller entity *e_a_* and the target entity *e_t_*. ${CF}_{e_{a}e_{t}}$ is computed as equation 1.

The Section: The Confidence Factor on Page12 is now revised as follows.

Here, the variable $n$ denotes the total number of unique sentences obtained from GIX predicting *e_a_e_t_*, i.e. the regulation between *e_a_* and *e_t_*. The variable $v_{s}$ is the RE classifier prediction of regulation *e_a_e_t_*, of the *s^th^* sentence. It is obtained as the Stage-2 output of the GIX and acquires a value between 0 and 1. The parameter $P_{e_{a}e_{t}}$ represents the prior knowledge about both the agent entity (*e_a_*) and its interaction (*e_a_e_t_*) with the target entity e_t_. The constant *M* is the total number of curated databases under consideration. *K* is a factor balancing the influence of term $\sum_{s=1}^{n} v_{s}$and $\sum_{T=1}^{M} P_{e_{a}e_{t}}^{T}$ (also referred to as ${CF}_{1}$ and ${CF}_{2}$). The discrete variable $P_{e_{a}e_{t}}$of Equation 2 can acquire three different values for three different conditions, namely, (i) If $e_{a}$ is known to be a controller gene and the relation $e_{a}e_{t}$ is unknown, $P_{e_{a}e_{t}}$=1; (ii) If $e_{a}e_{t}$ is a known relation, the value of $P_{e_{a}e_{t}}$ is given a higher value of 3 compared to (i) accounting for the presence of two entities and a connecting arc; (iii) For other conditions, $P_{e_{a}e_{t}}$=0.

**Query 10.** - *Line 356, it says “… are less likely to be true…” but this is the opposite as it is said before, having a higher CF gives more confidence in a relation, not lesser. Additional comment on CF. You should search for some ways to normalize this value, in other way there is no reference to which could be a good value of CF. It should be bounded between a minimum and a maximum known value. Otherwise you will find that for some particular use a CF=5 is indicative of a highly probable relation, and for another use the same value of CF=5 could indicate a low probability for a relation, which is very counterintuitive from the point of view of the user of your tool*.

**Response to Query 10:** We thank the reviewer for highlighting the inaccuracy of the statement in Line 356. We agree with the reviewer that a higher Confidence Factor (*CF*) imparts more confidence in the relationship. The statement has been corrected as follows:

Interactions confirmed from a higher number of experimental and/or analytical sources will have a higher ${CF}_{e_{a}e_{t}}$ and thus, are more likely to be true.

Normalisation:

Normalisation involves establishing both lower and upper bounds. The lower bound of the confidence factor (CF) is 0, while the upper bound depends on the number of publications related to the organism. Given that this value may increase over time, proposing a universal normalisation process could become challenging. Nevertheless, the unnormalized CF remains meaningful, as it offers a relative measure of confidence. For instance, when comparing two queries on the same dataset—query 1 with CF=6.8 and query 2 with CF=2.3—researchers can infer that the relationship extracted from the first query holds a higher level of confidence, supported by larger number of publications compared to the second query.

**Query 11.** *Line 364. The numbering of experiments is quite confusing, as they are numbers 1.3, 1.4 and 1.5 , while they are not in section 1, and there is not 1.1 nor 1.2. You should label clearly your experiments, and then USE THOSE LABELS when you explain the experiment. As it can be seen in your manuscript, there is no reference to these labels later when the experiment are really explained.*

**Response to Query 11**: We agree with this observation and regret the error. We have updated the paragraph containing line 364 and aligned the section headings with labels Exp1, Exp2, and Exp3. The references for these three experiments throughout the text are also updated.

The following revised text is inserted in Section: Results on Page 13.

We conducted three independent experiments to assess the effectiveness of GIX. The first experiment (Exp1) demonstrated the Relation Extraction Capability (Stage-2) of the GIX framework using four well known benchmark datasets for gene/protein interactions. The second experiment (Exp2) evaluated automated extraction using GIX against the manual curation of a benchmark dataset. The third experiment (Exp3) evaluated GIX against the manual curation of a real-world database of transcriptional regulations.

The labels for each experiment section are now as follows:

Exp1 - Relation extraction capability (Stage-2) of the GIX framework

Exp2 - Comparison of GIX with manual curation of a benchmark dataset

Exp3 - Comparison of GIX with manual curation of a real-world database

**Query 12.** *Line 383, section Selection of keywords: this procedure seems quite artisanal, needing manual intervention by the user. If you are going to use the most repeated keywords, you should automatically choose them, without having to curate them from a larger set.*

**Response to Query 12.** Indeed, the selection of keywords requires manual intervention. As carried out in our work, identifying the words as keywords (in the ‘Selection of keywords’ experiment) is to be done as a one-off activity without requiring any additional manual input. The keywords identified in the ‘Selection of keywords’ experiment are suggested to be directly used by researchers who want to extract regulatory interactions among genes for any organism, without requiring any additional manual input. In our opinion, automating the process of selecting keywords may not result in significant benefit. Moreover, designing an automated query system can prove challenging due to diverse database designs and the coding requirements which can eventually affect the overall accuracy. Hence, although the proposed approach may appear artisanal, it is simple and straightforward to implement and the researchers can easily extend it to other domains.

**Query 13.** Lines 442 to 445. *The values for precision, recall and f-score in table 5 are exactly the same for the two reported methods. I am not sure if this is an error or effectively both alternatives produce the same values for those indexes. If this is the case, the text should discuss more clearly this issue and explain it.*

**Response to Query 13:** We clarify that the identical performance for GIX-RE and GIX (full) is not an error. In fact, we anticipate them to be identical. This is because we aim to demonstrate that GIX, utilising a few target-related keywords (a simpler approach), instead of manually collecting, refining, and annotating sentences (more complicated and cumbersome approach), continues to maintain a same accuracy in extracting interactions without losing crucial information.

The following revised text is inserted in Section: Exp-2 Comparison of GIX with manual curation of a benchmark dataset on Page 19.

Table 5 (of the manuscript) displays the results of two experiments labeled as Exp2-(i) GIX-RE (only relation extraction model) and Exp2-(ii) GIX (Full Framework). In experiment Exp2-(i), only the relation extraction model of GIX, corresponding to Stage-2 of the framework, is utilised. The datasets used for fine-tuning of the relation classification model for experiment Exp2-(i) include HPRD50, IEPA, and BioInfer while LLL dataset is used for testing. In experiment Exp2-(ii), the full GIX framework is under investigation. Here, the fine-tuning datasets consist of HPRD50, IEPA, and BioInfer. While the testing dataset comprises of sentences extracted from PubMed related to *Bacillus Subtilis* regulatory interactions. These two experiments demonstrate that GIX, using just a few target-related keywords instead of the manually refined and formatted sentences was able to maintain a similar level of accuracy of extracted interactions without losing any of the sentences.

**Query 14.** *Line 445, figure 7: this figure shows the two terms used in calculation of CF. As already discussed, the values of CF should be normalized to a fixed range. And moreover, as can clearly be seen in figure 7, there is a very large difference in scale between both terms in equation 1, whit the second one clearly dominating by a large proportion over the first one. This makes the first term (which is the one were the proposed method contributes) completely useless, meaning that all the decisions will be directed by the previous knowledge, and in this way the full method is not taking any advantage from all the previously described work. What I mean is that if you completely remove the first term you can find relations and attribute them using the second, and the results will be no different of your proposal. In this way I think this experiment cannot demonstrate at all what it wanted to demonstrate.*

**Response to Query 14:** While we tend to loosely agree with the general arguments on the significance of the weights of the two components of CF, we have a diverging opinion on their conclusion related to the irrelevance of the first term – in our case. It may be noted that in calculating CF (Confidence Factor), CF_1_ serves as the baseline whereas CF_2_ is used to adjust the final CF value. It is important to note that CF_2_ depends on whether relations are available in manually curated public repositories. These databases primarily focus on well-studied organisms like *E. coli* or humans, therefore, relationships for less-researched organisms may not be available. Moreover, due to explosive growth in publications, not all the identified relationships get immediately recorded. For instance, in Exp2 for *Bacillus Subtilis*, out of 706 extracted interactions, only 160 have a value for CF_2_. Excluding CF_1_ would result in the loss of 546 relationships, which would be inappropriate . Therefore, it is crucial to include CF_1_.

However, considering the arguments presented by the reviewer, we have revised the Figure 7 (Figure 3 here), to illustrate the impact of the terms CF_1_ and CF_2_ on the overall confidence factor (CF).

Figure 3: Confidence factor ($\boldsymbol{CF}_{\boldsymbol{e}_{\boldsymbol{a}}\boldsymbol{e}_{\boldsymbol{t}}}$) of the extracted relations for *Bacillus subtilis* distribution of $\boldsymbol{CF}_{\boldsymbol{e}_{\boldsymbol{a}}\boldsymbol{e}_{\boldsymbol{t}}}$ with and without prior knowledge.

**Query 15.** *Line 498. In the experiment reported in this area, the idea if I am not wrong is to use the proposed method to curate a database of interactions. What I find challenging and unexplained is why you use the same RegulonDB you are trying to curate as prior knowledge for the curation process. One would have expected some external knowledge as prior, so you can include external information that can incorporate new knowledge. This is like making yourself a test for your knowledge on a subject, and instead of asking someone else to correct your test to see if you answered it well, correcting it yoursfcarself, with the same knowledge you used to answer it. Key finding, you will get an A+ score, which does not mean you have acquired the knowledge.*

**Response to Query 15:** We apologize for any confusion caused. In response to the reviewer, we clarify that the proposed algorithm does not curate biological relationships from RegulonDB. Instead, it curates a new database by extracting relations from PubMed abstracts. This new dataset utilises the known relations already existing in RegulonDB to validate the automatically extracted relationships (by the proposed algorithm) from the published literature. For instance, in the experiment (Exp3), 92% of the extracted relations had either the relationship or else, the presence of agent gene in RegulonDB. Therefore, to reiterate, the use of RegulonDB serves only to confirm the accuracy of the extracted relationships.

The following revised text is inserted in Section: Exp3- Comparison of GIX with manual curation of a real-world database on Page: 20.

The extracted relationships are validated using known relations from RegulonDB, ensuring accuracy, and the overall confidence factor is adjusted based on this ground truth for evaluating GIX-based relationships against manually curated ones by RegulonDB.

Reviewer 4

General:
***Query 1:*** *Overall text content should be revised, edited and shortened. Similarly the references which are relevant should be cited. Example: For such a very specific objective/aim of the paper, Introduction is too lengthy, more than 1400 words with 22 references. Introduction-to outline the Aims and Objectives of this paper for a reader.*

**Response to Query 1:** We appreciate your feedback into the need for revisions in the overall text content and the importance of citing relevant references. Our work systematically addresses distinct segments of relation extraction, ranging from data retrieval to the refinement of retrieved relations. This thorough exploration of each element contributes to the extended length, providing a detailed account of the complexities inherent in our approach. In response to your specific concern about the length of the introduction section, exceeding 1400 words, we have revised and edited the content. The introduction now succinctly outlines the aims and objectives of the paper within a reduced word count of 1001 words, while ensuring clarity and focus. Most of the relevant papers cited in the introduction section are focused on the challenges and methodologies in relation extraction from published literature using NLP models for biological genetic interactions. However, we agree that the list of cited references can be improved. Three additional papers have been included, highlighting the application of deep learning models for relation extraction [16, 17, 18] .

***Query 2.*** *Authors should keep in mind to revise to make each section concise and comprehensible.
Specific. Background literature should concentrate on current AI approaches which can facilitate data extraction beyond the Key words and subject headings(e.g. MeSH) for data mining.*

**Response to Query 2:** The text has been revised as requested. We have updated the manuscript to incorporate information on standardization efforts in medical vocabulary and the challenges posed by data retrieval amidst information overload in publications. In the context of current AI approaches, the section focuses on pre-trained large language models and Named Entity Recognition transformer-based models like Bert, ELMO, BioBERT, and BERN2, which are pre-trained on domain-specific literature and fine-tuned for specific tasks. The background preliminaries are tailored for a broad audience, covering unstructured text aspects related to relation extraction, essential dataset details for the experimental sections, and recent AI and relation extraction models.

***Query*** *3.* *Methods: should explicitly focus on pre-and post-processing, followed by the proposed confidence factor with examples from the published literature on the subject headings.*

**Response to Query 3:** As per the reviewers suggestion, we have revised the method section by offering a more thorough discussion of the tools and processes used in pre-processing, relation extraction, and post-processing. We have incorporated specific sections with example sentences to clarify procedural steps, improving the overall clarity and comprehensibility of the methodological details in response to this feedback.

***Query 4.*** *Results: the authors extend the argument with benchmark datasets and real-world database that GIX (Gene Interaction Extraction) should become the overarching approach. Is this the overall outcome derived?*

**Response to Query 4:** Experiments with benchmark datasets and real-world database demonstrate GIX's capacity to align with manual curation exhibiting enhanced robustness. This positions GIX as a compelling choice for biological relation extraction, potentially serving as the predominant solution. The growing volume of literature makes manual curation increasingly challenging. Although various NLP tools have been employed, they often fall short of completely eliminating manual intervention. GIX, on the other hand, delivers faster and reliable results, making it a practical alternative.

***Query 5.*** *Conclusion and Abstract should give homogenous statement (e.g. Sentence in Abstract, “With suitable experiments, we show GIX's capability to augment existing datasets with new sentences”). Thus GIX appears to be a tentative step.*

**Response to Query 5:** Thank you for pointing out the need for a homogeneous statement between the Conclusion and Abstract. We understand the importance of clarity in our work and have revised the abstract accordingly to align with the conclusion.

The following revised text has been inserted in section: Abstract on page 2

GIX's Stage-2 relation extraction method demonstrated superior performance on benchmark protein/gene interaction datasets using 10-fold cross-validation, surpassing state-of-the-art approaches. We demonstrate that the proposed method, although fully automated, performs as well as the manual relation extraction process, with enhanced robustness. We also observed GIX's capability to augment existing datasets with new sentences, incorporating newly discovered biological terms and processes. Further, we demonstrate GIX’s real-world applicability in inferring *E. coli* gene circuits.

# I. References

| [1] | scikit-learn., n.d.. [Online]. Available: https://scikit-learn.org/stable/modules/generated/sklearn.model_selection.KFold.html. [Accessed 2023 August 8]. |
| --- | --- |
| [2] | “Identifying protein-protein interactions in biomedical literature using recurrent neural networks with long short-term memory,” in *Proceedings of the eighth international joint conference on natural language processing (volume 2: short papers)*, 2017. |
| [3] | C. Quan, L. Hua, X. Sun and W. R. O. Bai, “Multichannel Convolutional Neural Network for Biological Relation Extraction,” *BioMed research international,* vol. 2016, p. 1850404–10, 2016. |
| [4] | A. Airola, S. Pyysalo, J. Björne, T. Pahikkala, F. Ginter and T. Salakoski, “All-paths graph kernel for protein-protein interaction extraction with evaluation of cross-corpus learning,” *BMC bioinformatics,* vol. 9, no. 11, pp. S2-S2, 2008. |
| [5] | Y. Zhang, H. Lin, Z. Yang and Y. Li, “Neighborhood hash graph kernel for protein–protein interaction extraction,” *Journal of biomedical informatics,* vol. 44, no. 6, pp. 1086-1092, 2011. |
| [6] | Y. Peng, S. Gupta, C. H. Wu and K. Vijay-Shanker, “An extended dependency graph for relation extraction in biomedical texts,” in *Proceedings of BioNLP 15*, 2015. |
| [7] | Y.-C. Chang, C.-H. Chu, Y.-C. Su, C. C. Chen and W.-L. Hsu, “PIPE: a protein-protein interaction passage extraction module for BioCreative challenge,” *Database : the journal of biological databases and curation,* vol. 2016, p. 101, 2016. |
| [8] | S. Kim, J. Yoon, J. Yang and S. Park, “Walk-weighted subsequence kernels for protein-protein interaction extraction,” *BMC bioinformatics,* vol. 11, no. 1, p. 107, 2010. |
| [9] | H. Zhang, R. Guan, F. Zhou, Y. Liang, Z.-H. Zhan, L. Huang and X. Feng, “Deep Residual Convolutional Neural Network for Protein-Protein Interaction Extraction,” *IEEE access,* vol. 7, p. 89354–89365, 2019. |
| [10] | H. Zhang, R. Guan, F. Zhou, Y. Liang, Z.-H. Zhan, L. Huang and X. Feng, “‘A protein-protein interaction extraction approach based on deep neural network,” *IEEE access,* vol. 7, p. 89354–89365, 2019. |
| [11] | Y. Zhang, H. Lin, Z. Yang, J. Wang, S. Zhang, Y. Sun and L. Yang, “A hybrid model based on neural networks for biomedical relation,” *Journal of biomedical informatics,* vol. 81, pp. 83-92, 2018. |
| [12] | M. Ahmed, J. Islam, M. R. Samee and R. E. Mercer, “Identifying Protein-Protein Interaction using Tree LSTM and Structured Attention,” in *2019 IEEE 13th international conference on semantic computing (ICSC)*, 2019. |
| [13] | Biopython, 2022. [Online]. Available: https://biopython.org/docs/1.76/api/Bio.Entrez.html. [Accessed 16 August 2023]. |
| [14] | B. Soup., 2023. [Online]. Available: https://www.crummy.com/software/BeautifulSoup/. [Accessed 6 August 2023]. |
| [15] | N. Hardeniya, J. Perkins, D. C. N. Joshi and I. Mathur, Natural Language Processing: Python and NLTK, Packt Publishing Ltd., 2016. |
| [16] | M. Sung, M. Jeong, Y. Choi, D. Kim, J. Lee and J. Kang, n.d.. [Online]. Available: http://bern2.korea.ac.kr/documentation. [Accessed 6 August 2023]. |
| [17] | W. Gu, X. Yang and M. Yang, “MarkerGenie: an NLP-enabled text-mining system for biomedical entity relation extraction,” *Bioinformatics Advances,* vol. 2, no. 1, 2022. |
| [18] | W. Zhou, K. Huang, T. Ma and J. Huang, “Document-Level Relation Extraction with Adaptive Thresholding and Localized Context Pooling,” in *In Proceedings of the AAAI conference on artificial intelligence*, 2021. |
| [19] | A. Akkasi and M.-F. Moens, “Causal relationship extraction from biomedical text using deep neural models: A comprehensive survey,” *Journal of biomedical informatics ,* vol. 119, p. 103820, 2021. |
| [20] | S. Yang, S. Yoo and O. Jeong, “DeNERT-KG: Named Entity and Relation Extraction Model Using DQN, Knowledge Graph, and BERT,” *Appl. Sci.,* vol. 10, p. 6429, 2020. |
| [21] | B. Zhu and J. Stülke, “SubtiWiki in 2018: from genes and proteins to functional network annotation of the model organism Bacillus subtilis,” *Nucleic Acids Research,* vol. 46, no. D1, p. D743–D748, 2018. |
| [22] | M. Miwa, R. Sætre, Y. Miyao and J. Tsujii, “Entity-Focused Sentence Simplification for Relation Extraction,” in *Proceedings of the 23rd International Conference on Computational Linguistics*, Coling 2010, 2010. |
| [23] | K. Fundel, R. Küffner and R. Zimmer, “RelEx—Relation extraction using dependency parse trees,” *Bioinformatics,* vol. 23, no. 3, pp. 365-371, 2007. |
| [24] | S. Jonnalagadda, L. Tari, J. Hakenberg, C. Baral and G. Gonzalez, “Towards Effective Sentence Simplification for Automatic Processing of Biomedical Text,” *arXiv preprint arXiv:1001.4277,* 2010. |
