# Supplementary material for: Large language model based framework for automated extraction of genetic interactions from unstructured data
Source: PLoS One. 2024 May 21;19(5):e0303231. doi: 10.1371/journal.pone.0303231 (PMC11108146; doi:10.1371/journal.pone.0303231)
Supplement: Attachment — Submitted filename: Response to Reviewers.docx [file pone.0303231.s003.docx]

**Manuscript ID:** PONE-D-23-31346

**Title:** Large language model-based framework for automated extraction of genetic interactions from unstructured data

**Authors:**

Jaskaran Gill, Madhu Chetty, Suryani Lim, Jennifer Hallinan

We thank the editor and reviewers for their valuable insights and recommendations. All the suggested changes which were requested by the reviewers have been incorporated into the revised manuscript, thereby significantly improving its quality.

**Response to Reviewers’ comments**

Reviewer 1

***Comments:*** *Authors have not submitted a detailed rebuttal letter and other required files. Therefore, this reviewer is unable to evaluate the revision.*

***Response:*** It appears that there might be an issue with Reviewer 1 accessing the submitted rebuttal letter for Round 1. We want to emphasise that we have addressed all 30 queries raised by the four reviewers in detail (16 pages) with Reviewer 2 confirming satisfaction with our responses. However, for the convenience of Reviewer 1, we are resubmitting the round 1 rebuttal letter as ‘Rebuttal Letter (Resubmit for Reviewer 1) - Previous Round.docx’ in Others.

Reviewer 2

***Comments:*** *Dear Authors, Thank you for correcting the manuscript and addressing all my points in it. I suggest that you include the main study design in the Methods or Results section in addition to Figure 1.[Fig 1: Schematic of fully automated relation extraction using an LLM based GIX framework. The white 233 blocks signify the processes, and the grey blocks indicate the tools utilised within those processes.].*

*In particular, in addition to the schematic representation of relation extraction, it would be great to schematically represent all parts of the study design of all computational experiments used in the study, including "real-world applicability in inferring E. coli gene circuits".*

***Response:*** Thank you for your constructive feedback and acknowledgment of the manuscript revisions. For demonstrating the real-world applicability of inferring E. coli gene circuits, we used *Cytoscape* (<https://cytoscape.org/>) to visualise the top 500 GIX-extracted transcriptional regulations of *E. coli* obtained from the experiment, Exp3. Thus, to illustrate the computational components of Relation Extraction experiments, we have incorporated schematic diagrams for the processes involved in experiments Exp1, Exp2, and Exp3 in Figure 7, 8 and 11 respectively. These diagrams are in addition to the schematic representation of GIX framework in Figure 1.

The following Figure 1 (Figure 7 in the manuscript) has been inserted in Section Exp1- Relation extraction capability (Stage-2) of the GIX framework on Page 16.

**Figure 1:** The 10-fold cross-validation process for evaluating the Relation Extraction capability (Stage-2) of the GIX. Here, each iteration consists of equally divided 10 folds of the dataset, where 9 folds (white blocks) are used for fine-tuning, and the 10th fold (grey block) is used for testing. The overall performance (ϴ) is obtained by averaging the performance of each iteration (ϴ_i_). The notations P, R and F represent Precision, Recall, and F-score.

The following Figure 2 (Figure 8 in the manuscript) has been inserted in Section Exp2- Comparison of GIX with manual curation of a benchmark dataset on Page 18.

**Figure 2:** GIX's extraction process for Bacillus subtilis relations illustrating outputs at each step (dark grey blocks) and inputs such as datasets, databases, or target-specific keywords (light grey blocks).

The following Figure 3 (Figure 11 in the manuscript) has been inserted in Section Exp3- Comparison of GIX with manual curation of a real-world database on Page 21.

**Figure 3:** GIX's extraction process for Escherichia coli relations illustrating the outputs at each step (dark grey blocks) and inputs such as datasets, databases, or target-specific keywords (light grey blocks).
